# Identification and Verification of Immune Subtype-Related lncRNAs in Clear Cell Renal Cell Carcinoma

**DOI:** 10.3389/fonc.2022.888502

**Published:** 2022-06-02

**Authors:** Zhifeng Wang, Zihao Chen, Tengyun Guo, Menglin Hou, Junpeng Wang, Yanping Guo, Tao Du, Xiaoli Zhang, Ning Wang, Degang Ding, Xiqing Li

**Affiliations:** ^1^Department of Urology, Henan Provincial People’s Hospital, Zhengzhou University People’s Hospital, Henan University People’s Hospital, Zhengzhou, China; ^2^Department of Urology, Southern Medical University, Guangzhou, China; ^3^Department of Neurosurgery, Daping Hospital, Army Medical University, Chongqing, China; ^4^Department of Oncology, Graduate School of Guilin Medical University, Guilin, China; ^5^Department of Pathology, Henan Provincial People’s Hospital, Zhengzhou University People’s Hospital, Henan University People’s Hospital, Zhengzhou, China; ^6^Department of Oncology, Henan Provincial People’s Hospital, Zhengzhou University People’s Hospital, Henan University People’s Hospital, Zhengzhou, China

**Keywords:** immune subtype, ccRCC, LncRNA, co-expression network, nomogram

## Abstract

**Background:**

According to clinical study results, immune checkpoint blockade (ICB) treatment enhances the survival outcome of patients with clear cell renal cell carcinoma (ccRCC). Previous research has divided ccRCC patients into immune subtypes with distinct ICB response rates. However, the study on the association between lncRNAs and ccRCC immune subtypes is lacking.

**Methods:**

Differentially expressed lncRNAs/mRNAs between two major immune subgroups were calculated. A weighted gene co-expression network analysis (WGCNA) was conducted to establish the lncRNA-mRNA co-expression network and select the key lncRNAs. Then, prognostic lncRNAs were selected from the network by the bioinformatics method. Next, the risk-score was estimated by lncRNA expression and their coefficients. Finally, a nomogram based on lncRNAs and clinical parameters was created to predict the prognosis of ccRCC.

**Results:**

LncRNAs and mRNAs associated with ccRCC immune subtypes were identified. The lncRNAs and mRNAs from a gene module closely linked to the immune subtype were used to construct a network. The KEGG pathways enriched in the network were related to immune system activation processes. These 8 lncRNAs (AL365361.1, LINC01934, AC090152.1, PCED1B-AS1, LINC00426, AC007728.2, AC243829.4, and LINC00158) were found to be positively correlated with immune cells of the tumor microenvironment. The C-index of the nomogram was 0.777, and the calibration curve data suggests that the nomogram has a high degree of discriminating capacity.

**Conclusion:**

In summary, we discovered core lncRNAs linked with immune subtypes and created corresponding lncRNA–mRNA networks. These lncRNAs are anticipated to have predictive significance for ccRCC and may provide insight into novel biomarkers for the disease.

## Introduction

RCC comprises 90 percent of renal tumors (1) and accounts for 3% of adult solid tumors (2). ccRCC is the most prevalent type of RCC, comprising about 80 percent of RCC individuals and having the highest fatality rate (3). In 2022, it is estimated that about 79,000 cases of RCC will be diagnosed in the USA, and about 14,000 patients will die from RCC, respectively (4). Age, smoking, hypertension, and obesity are the most well-known risk factors for RCC (5). There is a range of approved therapy options for ccRCC, including surgical resection, targeted therapy, and new immunotherapy drugs. Despite these therapies, around 50% of ccRCC patients acquire metastatic disease, and their survival rate remains below 10% over five years (6). The key to developing individualized therapy and determining the prognosis of ccRCC is the identification of biomarkers.

LncRNAs (long noncoding RNAs) are RNAs that surpass 200 nucleotides in length but are not capable of coding proteins (7). LncRNA can influence gene transcription and translation by multiple processes, including chromosomal remodeling and protein inhibition. Recent data suggests that lncRNAs perform a significant role in tumorigenesis and cancer outcomes (8, 9). For example, in recent research, LUCAT1 was shown to be a poor prognosis predictive factor in ccRCC (10). More than 50,000 lncRNAs have been discovered on human chromosomes. However, the function of these lncRNAs is unclear.

Because ccRCC demonstrates high levels of heterogeneity (11), it is challenging to anticipate overall survival and develop appropriate therapeutic approaches. Several earlier studies have explored the classification of ccRCC individuals into subgroups based on their genetic features (12, 13). For instance, in our prior research, we found two unique immune subtypes of ccRCC, each with a distinct clinical prognosis (14). The subtype with immune cells had a worse prognosis with surgical treatment but a better prognosis with immunotherapy treatment. However, the analysis of lncRNAs with ccRCC immune subtypes is lacking. Thus, discovering subtype-related lncRNAs as prognostic biomarkers for ccRCC patients and then developing personalized therapy regimens for ccRCC patients is critical and promising.

To predict the outcomes of ccRCC patients, we created a model using eight prognostic lncRNAs and clinical data. The expression data was evaluated to detect subtype-related differentially expressed lncRNAs. A regulatory connection network comprised of co-expressed mRNAs and lncRNAs was created using WGCNA. Then, using the least absolute shrinkage and selection operator (LASSO) approach, prognostic lncRNAs were picked from the network. Following that, we estimated the risk score by multiplying the lncRNA expression and their coefficients. Finally, a nomogram was constructed using lncRNAs and clinical parameters to predict the prognosis of ccRCC.

## Materials and Methods

### Data Collection

The TCGA database was selected to obtain gene expression data and relevant clinical details for patients with ccRCC. Since the expression profiles were accessible, a total of 611 ccRCC samples were considered for additional investigation. Of the 611 samples, 539 were ccRCC samples and 72 were normal samples. This cohort consists of 534 samples with available clinical parameters, comprising of 344 males (64%) and 190 females (36%). The median age of this cohort is 61, and it ranges from 26 to 90. In total, 47% (252/534) of the tumors were located on the left, and 53% (281/534) of the tumors were located on the right. According to the Cancer Staging Manual, there were 268 stage(I), 57 stage(II), 123 stage(III), and 83 stage(IV) ccRCC samples.

We collected level3 expression profiles (FPKM, fragments per kilobase of transcript per million fragments mapped) of these KIRC samples. Then, the FPKM profiles were converted into transcripts per million (TPM). Annotations of the 19196 mRNAs and 14042 lncRNA transcripts were conducted by “Homo_sapiens.GRCh38.105.gtf” and ‘gencode.v35.long_noncoding_RNAs.gtf’, respectively. When several probes relate to the same gene, the maximum expression of the gene is chosen.

### Differentially Expressed mRNAs and lncRNAs

In order to find the DEMs and DELs, the normal kidney samples were removed. Then, the program edgeR was used to filter DEMs and DELs (15). Subtype1 and subtype2 information came from our previously published article (14). In total, 301 subtype1 and 229 subtype2 samples were used in the process of differential expression analysis. In the DELs analysis, |log2FoldChange|>0.2 and a p-value of 0.05 were used to determine the screening criteria. In the DEMs analysis, |log2FoldChange|>0.5 and a p-value of 0.05 were used to determine the screening criteria. The cutoff values for DELs and DEMs analysis were different since the average expression values of lncRNAs were low and fewer lncRNAs could be identified as DELs.

### Co-Expression Analysis

Using WGCNA, the co-expression patterns of DELs and DEMs between subtype1 and subtype2 samples were determined. The “WGCNA” R package was used to generate a co-expression network using the DEL and DEM expression profiles (16). (1) Outlier samples were removed. (2) A weighted adjacency matrix was built by a soft-thresholding parameter β. (3) Following that, the adjacency matrix was transformed to the topological overlap matrix (TOM), and the hierarchical clustering using the TOM-based dissimilarity was done. (4) Additionally, RNAs were classified into several modules by their TOM dissimilarity. (5) We calculated correlations between modules and clinical characteristics.

### Establishing a LncRNA-mRNA Co-Expression Network

Pearson correlation analysis was conducted for the construction of the co-expression network of lncRNAs/mRNAs. The co-expression pairs with p-value<0.01 and correlation>0.8 were retained. The co-expression network was plotted by the retained co-expression pairs and the Cytoscape application. We performed enrichment analysis for mRNAs from the network using the clusterProfiler R package (17). Enriched pathways with a p-value<0.05 were regarded as statistically significant.

### Development of a Survival Prediction Model by LncRNAs and Age

LASSO is a prevalent method for selecting lncRNAs from high-dimensional predictors. The present research used the ‘glmnet’ package to conduct the LASSO regression analysis (18). Then, using multivariate Cox regression analysis, a risk score model containing age and eight lncRNAs was constructed. The following mathematical model was used to generate the risk score: risk score = Coef(Gene1)*x(Gene1)+ … + Coef(GeneN)*x(GeneN), where Coef (GeneN) and x(GeneN) denote the coefficient value and lncRNA expression values, respectively. All ccRCC patients were split into high- and low-risk groups based on the median value. The mortality profiles of these 2 groups of samples were analyzed using log-rank testing. The diagnostic effectiveness of the model was determined using area under curves (AUC).

### Estimation of Immune Infiltration and Establishing of Nomogram

We assessed immune infiltration using the MCP-counter approach. The “MCP-counter” software generates relative presence scores for 10 immune cell and stromal cell types using normalized expression (19). To examine the 1-, 3-, and 5-year OS of patients, we created a nomogram by merging clinical factors such as age, stage, and lncRNAs. The concordance index (C-index) was then used to assess the nomogram’s prediction performance. Additionally, three calibration curves for 1, 3, 5 years were created to assess the consistency between predicted and observed survival.

### RNA Preparation and Reverse Transcription

We collected 10 cases of normal renal tissue (suspected nephritis or biopsy confirmed normal tissue) and 20 cases of renal cell carcinoma. The tissue samples stored at -80°C were loaded into RNase-free mortar, and 0.1 g tissue was quickly added with 100 ul Trizol for grinding into homogenate, then added with 400 ul Trizol for blending, and stood at room temperature for 5 minutes. RNA was prepared according to the manufacturer’s instructions. The obtained RNA was dissolved in 20 ul of RNase-free water, and 2 ul was taken to measure the RNA concentration by a micro UV-Vis fluorescence spectrophotometer (e-spect, Malcom, Japan).

RNA reverse transcription was performed using a reverse transcription kit,2 ul of 5×gDNA Eraser Buffer, 1ul of gDNA Eraser and 7 ul of RNA were gently mixed evenly, 1 μL of total RNA, 4 μL of 5 × buffer, 1 μL of RNase inhibitor, 2 μL of dNTPs, and 1 μL of reverse transcriptase. Reaction parameters were 42°C for 60 min and 95°C for 5 min. The obtained cDNA was preserved at -80°C for further use. The obtained cDNA is stored at -80°C for further use.

### Quantitative Polymerase Chain Reaction

qPCR was reacted in a 20-μL system containing 5.6 ul of cDNA, 2 × Master Mix with 0.03 × ROX added, 2 ul of PCR Forward Primer(10×) and 2 ul of PCR Reverse Primer(10×) on a Mx3005p cycler. PCR was amplified in triplicate and the cycling parameters were 95°C for 300 sec, followed by 40 cycles of 95°C for 15 sec and 60°C for 30 sec. The primer sequences are listed in **Table 1**.

**Table 1:** Primers used for quantitative polymerase chain reaction.

Primer	Sequence
**LINC01934**	
Forward	5’-GCTTTGCCAAGCTCAGTTCC-3’
Reverse	5’-TGTTGGTCTCTCAGTTTAGGATGA-3’
**LINC00158**	
Forward	5’-CTGGTTGAATTGAATGTGAAGAGGA-3’
Reverse	5’-TGGAGCTGCTGGAGAAAAACA-3’
**AC007728.2**	
Forward	5’-CCTTAGGCAACACCGTTCTCA-3’
Reverse	5’-GCTTTCCCCATGTCTCGACT-3’
**GAPDH**	
Forward	5’-GTCAACGGATTTGGTCTGTATT-3’
Reverse	5’-AGTCTTCTGGGTGGCAGTGAT-3’

### Statistical Analysis

The expression levels of LncRNA in tissues were expressed as mean ± SD and compared with a two-sample t-test. Statistical analyses were performed using SPSS13.0. P<0.05 was considered statistically significant.

## Results

### Differential Expression of lncRNAs and mRNAs

**Figure 1:**
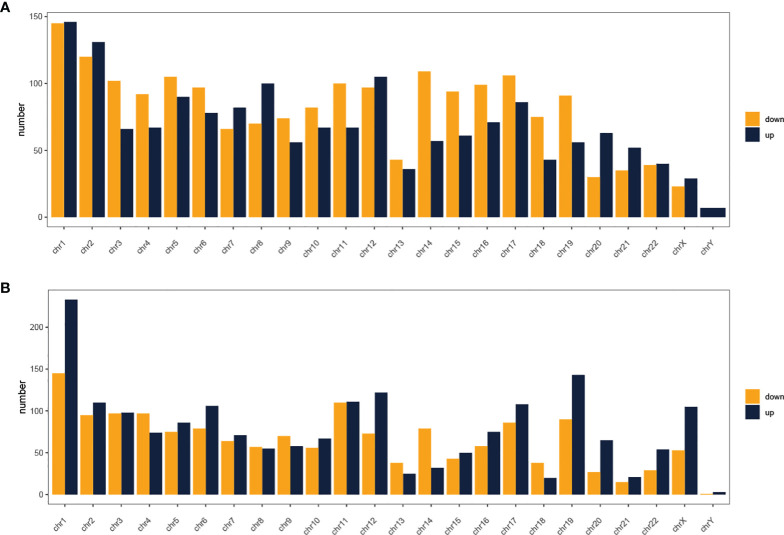
Distribution of dysregulated lncRNAs **(A)** and mRNAs **(B)** on human chromosomes. Yellow and blue indicate RNAs that are up- and down-regulated in subtype2 patients, respectively. Chr, Chromosome.

In total, using a logFC>0.2 and p-value<0.05 as the cutoff criteria, 3568 DELs between two subtypes were found, including 1667 upregulated and 1901 downregulated lncRNAs in subtype2. **Table 2** listed the top ten most up- and down-regulated lncRNAs. The distribution of DELs on human chromosomes was illustrated in **Figure 1A**. Similarly, using a logFC>0.5 and p-value<0.05 as the cutoff criteria, 3467 DEMs between two subtypes were identified, including 1892 upregulated and 1575 downregulated mRNAs in subtype2. The top 10 upregulated and downregulated mRNAs were shown in **Table 3**. The distribution of DEMs on human chromosomes was illustrated in **Figure 1B**. The expression patterns of DELs (**Supplementary Figure 1A**) and DEMs (**Supplementary Figure 1C**) were shown by heatmaps. Volcano plots were plotted to show the DELs (**Supplementary Figure 1B**) and DEMs (**Supplementary Figure 1D**).

**Table 2:** The top 10 DELs between two subtypes.

lncRNAs	logFC	logCPM	PValue	FDR
**ZNF350-AS1**	-2.45	8.41	<0.01	<0.01
**AC129507.4**	-2.96	7.59	<0.01	<0.01
**AP001628.2**	-1.94	7.07	<0.01	<0.01
**AC148477.4**	-3.24	7.99	<0.01	<0.01
**AP000757.1**	-1.80	8.34	<0.01	<0.01
**PCED1B-AS1**	1.20	9.44	<0.01	<0.01
**AC004585.1**	1.64	8.00	<0.01	<0.01
**USP30-AS1**	1.41	8.96	<0.01	<0.01
**LINC02084**	1.40	7.89	<0.01	<0.01
**LINC02528**	2.12	7.14	<0.01	<0.01

logFC, log2 fold change between the groups; logCPM, the average log2-counts-per-million; PValue, the two-sided p-value; FDR: adjusted p-value.

**Table 3:** The top 10 DEMs between two subtypes.

lncRNAs	logFC	logCPM	PValue	FDR
**NDRG2**	-1.0	6.00	<0.01	<0.01
**TMEM38A**	-2.31	4.34	<0.01	<0.01
**ALB**	-5.18	7.29	<0.01	<0.01
**CYP17A1**	-3.92	4.28	<0.01	<0.01
**AQP6**	-7.19	5.61	<0.01	<0.01
**AIM2**	2.09	2.99	<0.01	<0.01
**LAG3**	2.24	4.26	<0.01	<0.01
**GBP5**	1.91	4.51	<0.01	<0.01
**FCGR1A**	1.47	4.00	<0.01	<0.01
**SLAMF7**	1.74	4.53	<0.01	<0.01

logFC, log2 fold change between the groups; logCPM, the average log2-counts-per-million; PValue, the two-sided p-value; FDR, adjusted p-value.

### Identification of WGCNA Modules

**Figure 2:**
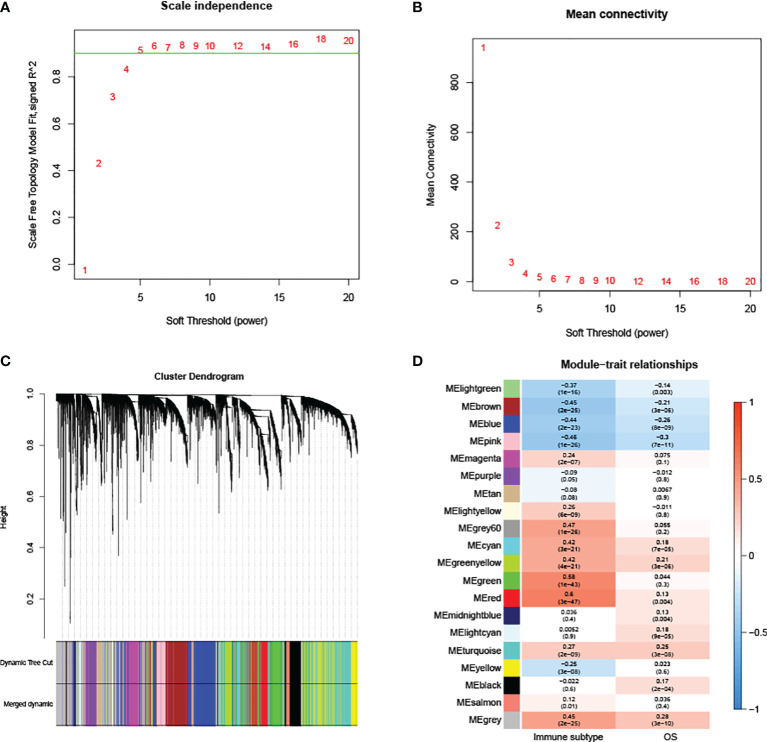
WGCNA analysis. **(A, B)** Scale independence and mean connectivity are used to determine the appropriate soft thresholding power. **(C)** Each branch corresponds to a particular RNA, and each color indicates a unique module made of co-expressed RNAs. **(D)** Correlations between RNA modules and clinical parameters, including immune subtypes and overall survival (OS). The MEred module was chosen for further analysis.

The expression profiles of 3467 DEMs and 3568 DELs were retained for the construction of the co-expression module. After the removal of outlier samples, 470 KIRC samples were kept (**Supplementary Figure 2**). A scale-free network was found when the soft-threshold power (β) value was set as ‘5’ (**Figures 2A, B**). The scale-free topology was plotted with R^2 =^ 0.9 and slope =−1.56 (**Supplementary Figure 3**). Having generated and merged close modules, a total of 20 modules were obtained (**Figure 2C**). The links between the generated modules and clinical characteristics have been illustrated (**Figure 2D**). For immune subtypes, the red module with 401 genes was selected since it had the most significant correlation.

### The lncRNA-mRNA Co-Expression Network

**Figure 3:**
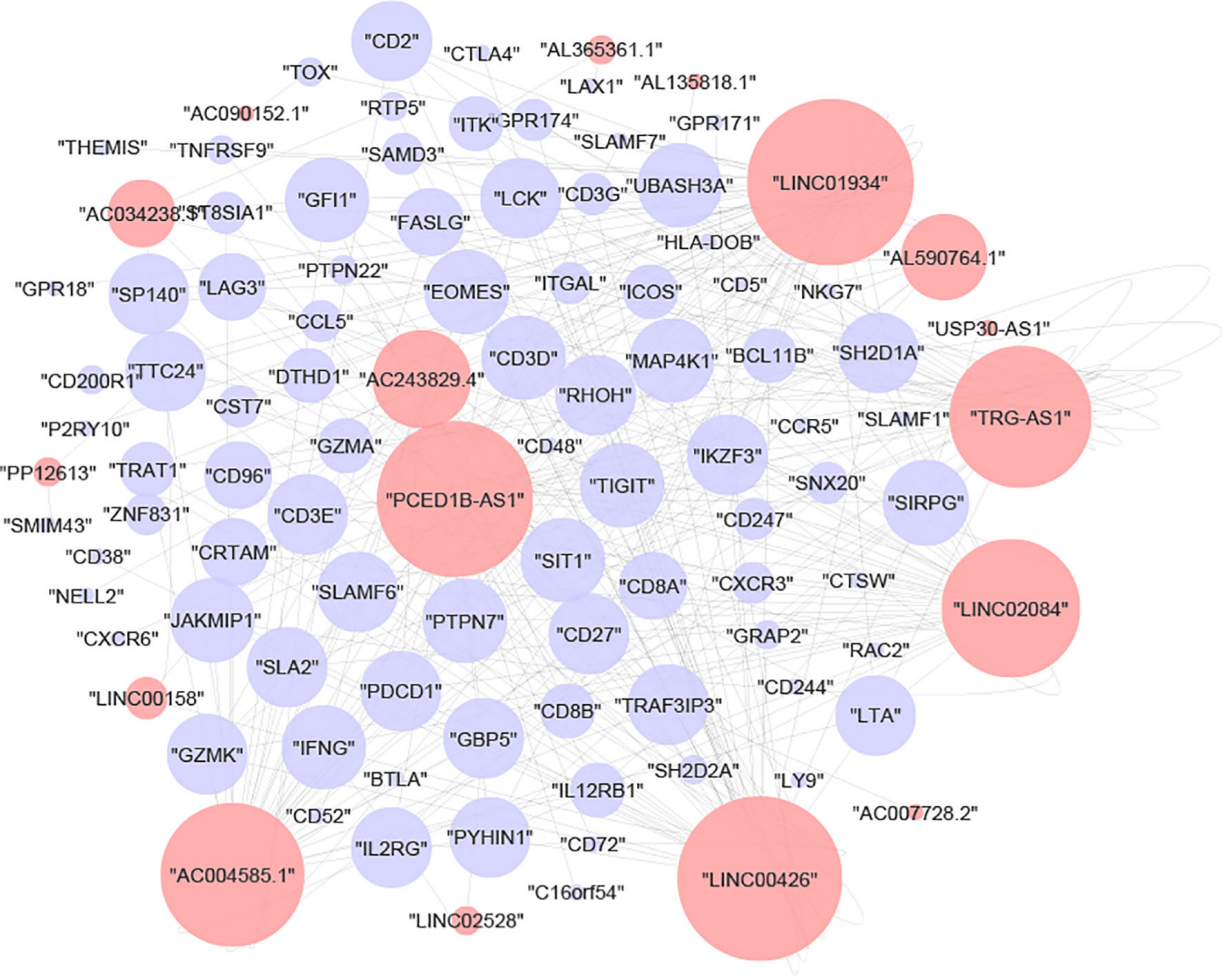
The lncRNA–mRNA co-expression networks. Red represents lncRNAs and blue represents mRNAs.

To deduce the hidden relationships between lncRNAs and mRNAs associated with immunological subtypes, the lncRNA-mRNA network was created using the chosen module’s lncRNA and mRNA expression profiles. After a strict screening process (correlation> 0.8 and p-value<0.05), 315 interaction pairs of lncRNAs/mRNAs (17 lncRNAs and 85 mRNAs) were plotted in Cytoscape (**Figure 3**). We then performed GO and KECG enrichment analyses on 85 mRNAs. We found that the immune-related pathways ‘T-cell-receptor-signaling-pathway’, ‘Primary-immunodeficiency’, ‘Th1-and-Th2-cell-differentiation’ (**Supplementary Table 1**) and immune-related items ‘T-cell-activation’, ‘regulation-of-T-cell-activation’, and ‘T-cell-differentiation’ (**Supplementary Table 2**) were enriched.

### Construction and Validation of Prognostic Models

**Figure 4:**
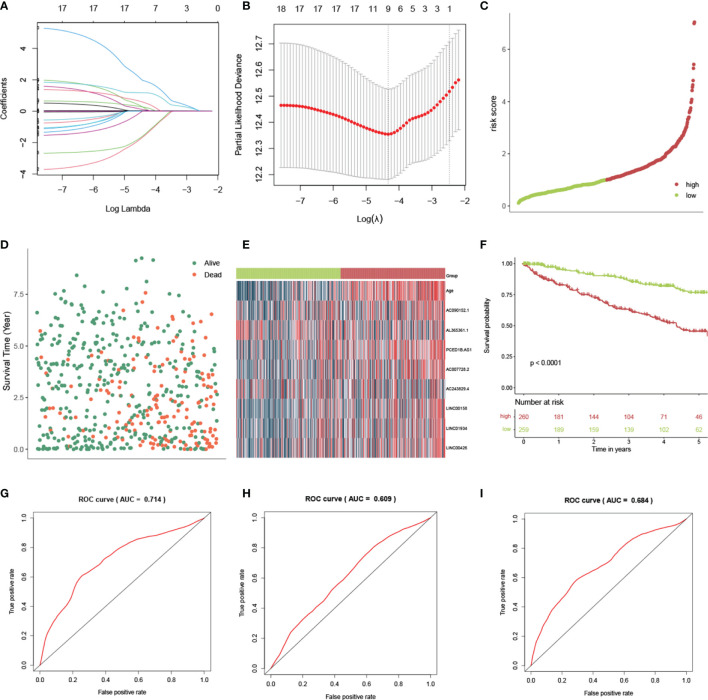
Construction and validation of the risk score by lncRNAs in ccRCC patients. **(A)** LASSO coefficient profiles of the lncRNAs. **(B)** The selection of the best lambda value. **(C)** The scatter plots of risk scores by 8 lncRNAs. **(D)** Survival overview and **(E)** heatmap for patients in the high- and low-risk groups. **(F)** The Kaplan-Meier (KM) curves demonstrate that patients with a low risk score have a much longer survival period than those with a high risk score. ROC curves show the accuracy of the combination of age and lncRNAs **(G)**, only age **(H)**, and only lncRNAs **(I)** in estimating the outcome of ccRCC patients from the TCGA database.

LASSO was conducted to select the important variables from 17 lncRNAs and age (**Figures 4A, B**). From this, 9 variables were identified, which included age and 8 lncRNAs (AL365361.1, LINC01934, AC090152.1, PCED1B-AS1, LINC00426, AC007728.2, AC243829.4, and LINC00158). The risk scores were estimated using the coefficients from the multivariate Cox regression model analysis. It was calculated as follows: risk score = 0.031*Age + (-3.24)*AL365361.1 + 1.33*LINC01934 + 0.67*AC090152.1 + 2.57*PCED1B-AS1 + 0.32*LINC00426 + 1.27*AC007728.2 + (-3.19)*AC243829.4 + 1.23*LINC00158). Following that, based on the median value, patients were classed as high- or low-risk. The relationships between risk score, survival time, survival status, and expression signatures of the 8 lncRNAs are shown (**Figures 4C** **–E**). Meanwhile, we compared OS across the two groups (**Figure 4F**) and discovered that OS was higher in the low-risk group (p-value<0.001). Using a ROC curve, the AUC value for the risk signatures, including age and 8 lncRNAs, was 0.714. (**Figure 4G**). AUC values for age and 8 lncRNAs were 0.609 (**Figure 4H**) and 0.684 (**Figure 4I**), respectively. Thus, the risk score will reach the highest AUC value by the combination of age and 8 lncRNAs. The risk score was able to predict health outcomes for ccRCC patients and was accurate at discriminating patient prognosis.

### The Diversification in Expression of lncRNAs

**Figure 5:**
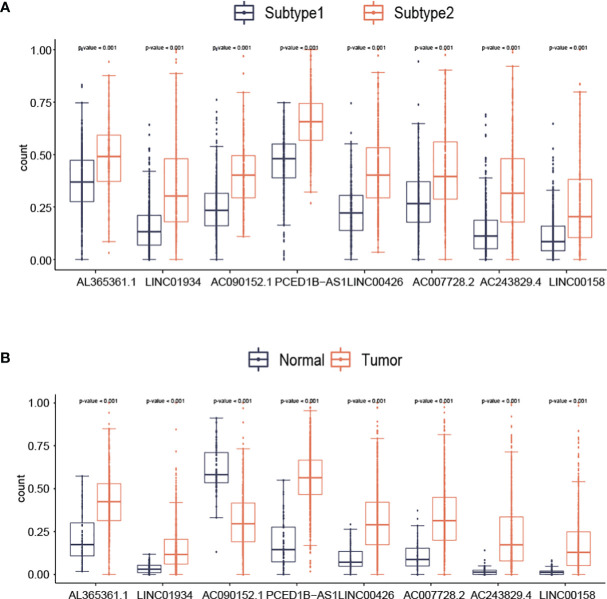
The diversification in expression values of lncRNAs among Subtypes **(A)** and Groups **(B)**.

With the use of boxplots, the diversification of 8 lncRNAs (AL365361.1, LINC01934, AC090152.1, PCED1B-AS1, LINC00426, AC007728.2, AC243829.4, and LINC00158) expression in two subtypes was ascertained. **Figure 5** suggests that the overall expression trend of lncRNAs was reported to be remarkably higher in subtype2 samples than in subtype1 samples (p-value<0.001; **Figure 5A**). For AL365361.1, LINC01934, PCED1B-AS1, LINC00426, AC007728.2, AC243829.4, and LINC00158, their expression values were higher in tumor samples than in normal samples (**Figure 5B**). For AC090152.1, its expression values were higher in normal samples than in tumor samples (**Figure 5B**).

**Figure 6:**
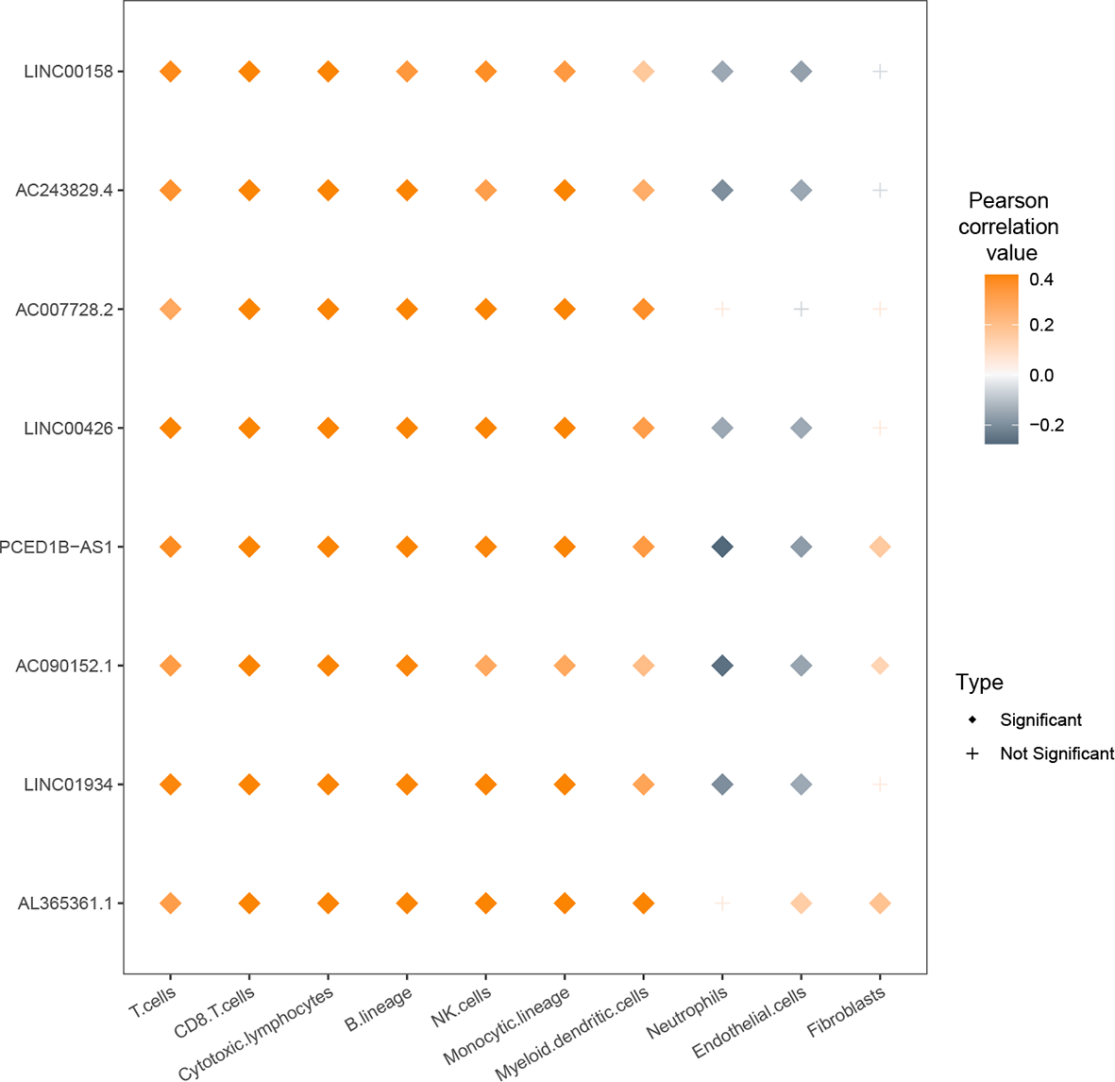
Relationship between expression values of nine lcnRNAs and infiltration of cells from the tumor microenvironment (TME).

We also correlated the lncRNA expression values with the cells from tumor microenvironment (TME). There were positive correlations between eight lncRNAs and populations of immune cells such as T cells (**Figure 6**). For LINC01934, AC090152.1, PCED1B-AS1, LINC00426, AC243829.4, and LINC00158, their expression values were negatively correlated with neutrophils and endothelial cells. For AL365361.1, AC090152.1, and PCED1B-AS1, their expression values were positively correlated with fibroblasts.

### Risk Score Correlated With Clinicopathological Factors

**Figure 7:**
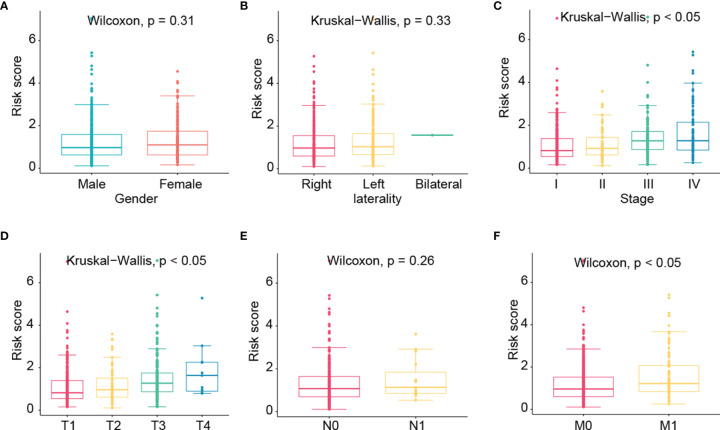
The correlation of risk score with clinicopathological factors such as gender **(A)**, laterality **(B)**, stage **(C)**, T **(D)**, N **(E)**, and M **(F)** groups.

We then further evaluated the risk scores among different genders, lateralities, stages, T, N, and M groups (**Figure 7**). No significant correlations were found in gender, laterality, and N groups. Besides, risk scores were found to be higher in advanced stage, T, and M groups.

### Establishing of Nomogram

**Figure 8:**
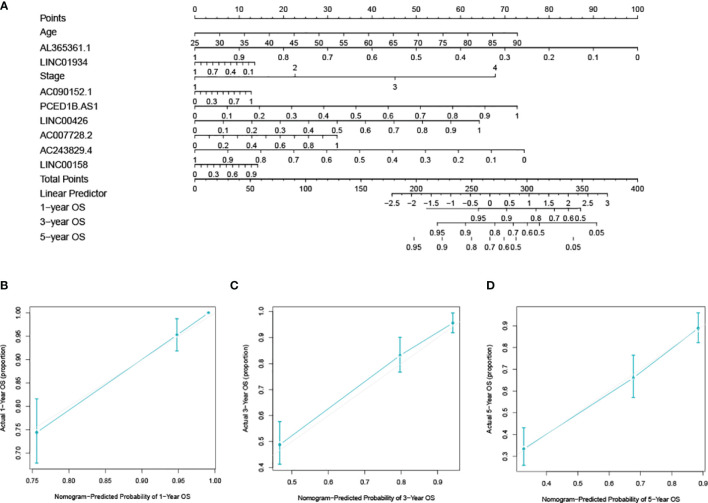
Developing and verifying a nomogram for prognosis prediction in the ccRCC dataset. **(A)** The nomogram was constructed based on age, stage, and lncRNAs. The calibration plots for predicting 1- **(B)**, 3- **(C)** and 5-year **(D)** survival.

The nomogram was created by integrating values about the age, stage, and the 8-lncRNA (**Figure 8A**). Calibration plots for 1-year, 3-year, and 5-year prognosis revealed no significant difference between nomogram predictions and actual observations (**Figures 8B** **–D**). The C-index was 0.777, and the calibration curve result indicates that the nomogram has a high degree of discrimination ability.

### Validation of Expression of lncRNAs

**Figure 9:**
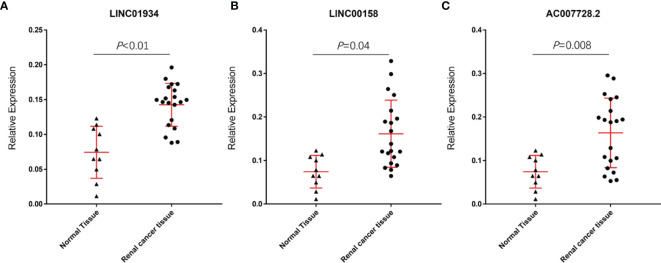
Relative expression values of LINC01934 **(A)**, LINC00158 **(B)**, and AC007728.2 **(C)** were significantly higher in renal cancer tissue than in normal tissue.

The results showed the relative expression values of LINC01934, LINC00158, and AC007728.2 in renal cancer tissue were substantially greater than in healthy tissue. (p-value< 0.05; **Figures 9A** **–C**). The research revealed no statistically significant variations in GAPDH expression, the Ct value of LncRNA was significantly lower in renal cancer tissues than in normal renal tissues, suggesting that LncRNA expression was upregulated in renal cancer.

## Discussion

The ccRCC is the most prevalent and fatal form of renal carcinoma. Though several therapeutic therapies for ccRCC have been established, the unsatisfactory mortality rate and resistance to chemotherapeutics create a rising need for novel therapeutic targets and predictive indicators to enhance clinical outcomes. Previous research has demonstrated that there are two ccRCC immune subtypes with distinct prognoses. However, little research has examined the prognostic relevance and functions of lncRNAs linked with immune subtypes.

In the previous study, 831 ccRCC samples were clustered into s1 and s2 by the scores of immune cells (14). Among these two subtypes, patients classified as s1 had a favorable prognosis than those classified as s2. In s2, immunotherapy biomarkers such as T cells were considerably increased. As a result, patients classified as s2 were recommended to get ICB therapy.

KEGG analysis demonstrated that the lncRNAs from the network were associated with immune signaling pathway, including ‘T-cell-receptor-signaling-pathway’ and ‘Th1-and-Th2-cell-differentiation’. The co-expression network analysis revealed that these lncRNAs were involved in immune cell infiltration. For example, there were substantial positive associations between the expression levels of eight lncRNAs and immune cell types such as T cells. These results suggested that these lncRNAs could influence the development and prognosis of ccRCC by regulating the immune cells in the TME.

Currently, building nomograms to predict the prognosis of cancers is prevalent (20). In a previous study, based on the hypoxia-associated lncRNAs and clinical parameters, a nomogram to assess 5-,7-, and 10- years prognosis of ccRCC was constructed (21). Time-dependent ROC curves showed AUC values of 5-, 7-, and 10- years were 0.604, 0.608, and 0.769, respectively. Based on LASSO analysis, we used eight lncRNAs from the lncRNA-mRNA network and then created a risk score model. Previous research has described several ccRCC prognostic models and nomograms based on lncRNAs, but our model had the following advantages: (1) The lncRNAs were filtered by DEG analysis and WGCNA, which guaranteed that these lncRNAs were DEGs of tumor/normal samples and immune subtype associated genes. (2) The C-index value of the model for predicting OS was 0.777. These features guaranteed the model’s reliability and hence increased its clinical application feasibility.

Among the 8 lncRNAs, LINC01934, AC090152.1, PCED1B-AS1, LINC00426, AC007728.2, and LINC00158 have negative values for coefficients. This result suggested that these lncRNAs were correlated with a negative prognosis. Similarly, AL365361.1 and AC243829.4 were associated with better prognosis. Currently, no study has been conducted to determine the role of these lncRNAs in ccRCC, thus this is the first research to demonstrate that increased AC007728.2, LINC00158, and LINC01934 are related to poor outcomes in ccRCC patients. However, additional fundamental research is required to discover their molecular roles in the progression of ccRCC.

However, this study has some limitations. First, although we established the lncRNA-mRNA network, we have not directly validated their regulatory correlation. Additional investigations are required to demonstrate the function of lncRNAs in ccRCC. Besides, other crucial clinical parameters such as pathological stages were not included in the constructed model. These clinical parameters could improve the accuracy of our model.

## Conclusion

This research has shown that lncRNAs and mRNAs engaged in the co-expression network of immune subtypes may serve as potential indicators. These newly identified lncRNAs demonstrated significant and positive correlations with immune cells in the TME. A risk score model and a nomogram for predicting prognosis were provided in this study. We expect that our study will contribute to the development of individual therapy.

## Data Availability Statement

The original contributions presented in the study are included in the article/**Supplementary Material**. Further inquiries can be directed to the corresponding authors.

## Ethics Statement

The data used in this study were extracted from publicly available database, strictly following the publication guidelines. Therefore, there was no requirement for ethics committee approval.

## Author Contributions

ZW, DD, and XL designed and conceived this project. ZW, ZC, TG, and MH analyzed the data. JW, YG, and TD plotted the figures. ZW, XZ, NW, DD, and XL wrote and revised the manuscript for important intellectual content. All authors read and approved the final manuscript.

## Funding

The project was supported by Application of the Fourth Generation Da Vinci Robot (Xi) and Mixed Reality Technology in Nephron Sparing Surgery for Complex Hilar Tumor (Science and Technology Research of Henan Provincial Health and Health Commission SBGJ202102020).

## Conflict of Interest

The authors declare that the research was conducted in the absence of any commercial or financial relationships that could be construed as a potential conflict of interest.

## Publisher’s Note

All claims expressed in this article are solely those of the authors and do not necessarily represent those of their affiliated organizations, or those of the publisher, the editors and the reviewers. Any product that may be evaluated in this article, or claim that may be made by its manufacturer, is not guaranteed or endorsed by the publisher.

## Supplementary Material

Supplementary Figure 1 Differentially expressed lncRNAs (DELs) and mRNAs (DEMs). Volcano plots show the DELs **(A)** and DEMs **(C)**. The colors red and blue denote up- and down-regulated expression, respectively. Heatmaps of DELs **(B)** and DEMs **(D)**.

Supplementary Figure 2 The outliers were removed before the WGCNA analysis.

Supplementary Figure 3 The verification of the constructed scale-free network. R2 = 0.9; slope=-1.56.
