# Supplementary figures and images for: Identification and Verification of Immune Subtype-Related lncRNAs in Clear Cell Renal Cell Carcinoma
Source: Front Oncol. 2022 Jun 2;12:888502. doi: 10.3389/fonc.2022.888502 (PMC9200973; doi:10.3389/fonc.2022.888502)

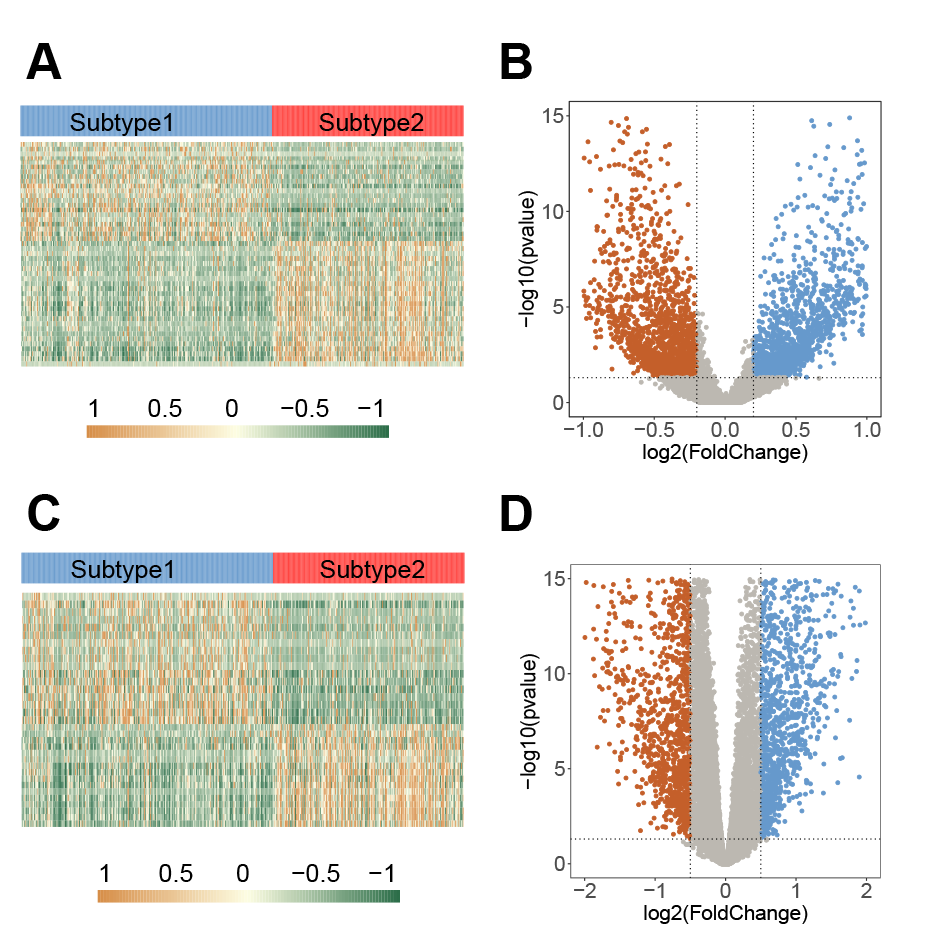

Supplement: Supplementary Figure 1 — Differentially expressed lncRNAs (DELs) and mRNAs (DEMs). Volcano plots show the DELs (A) and DEMs (C). The colors red and blue denote up- and down-regulated expression, respectively. Heatmaps of DELs (B) and DEMs (D). [file Image_1.tif]

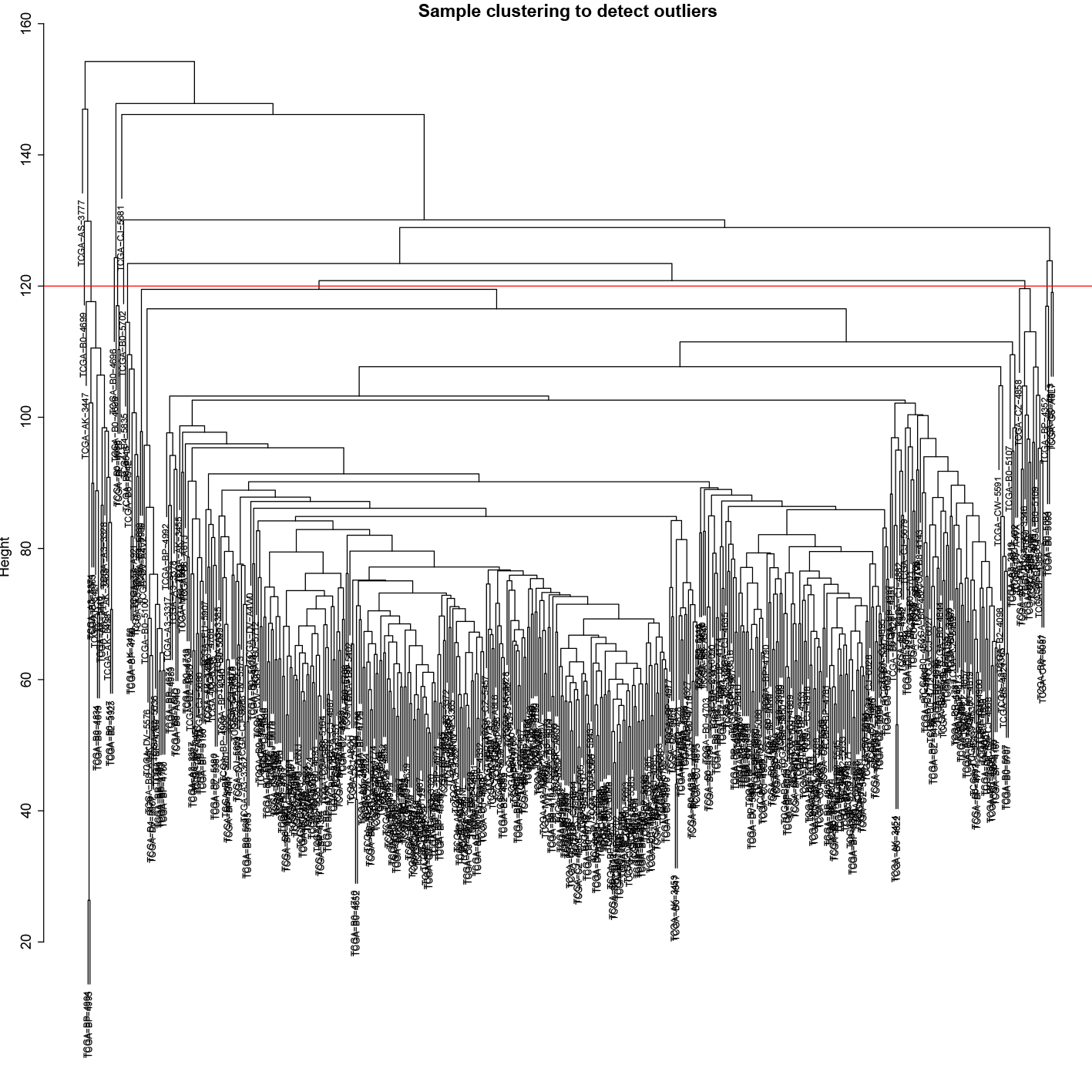

Supplement: Supplementary Figure 2 — The outliers were removed before the WGCNA analysis. [file Image_2.tif]

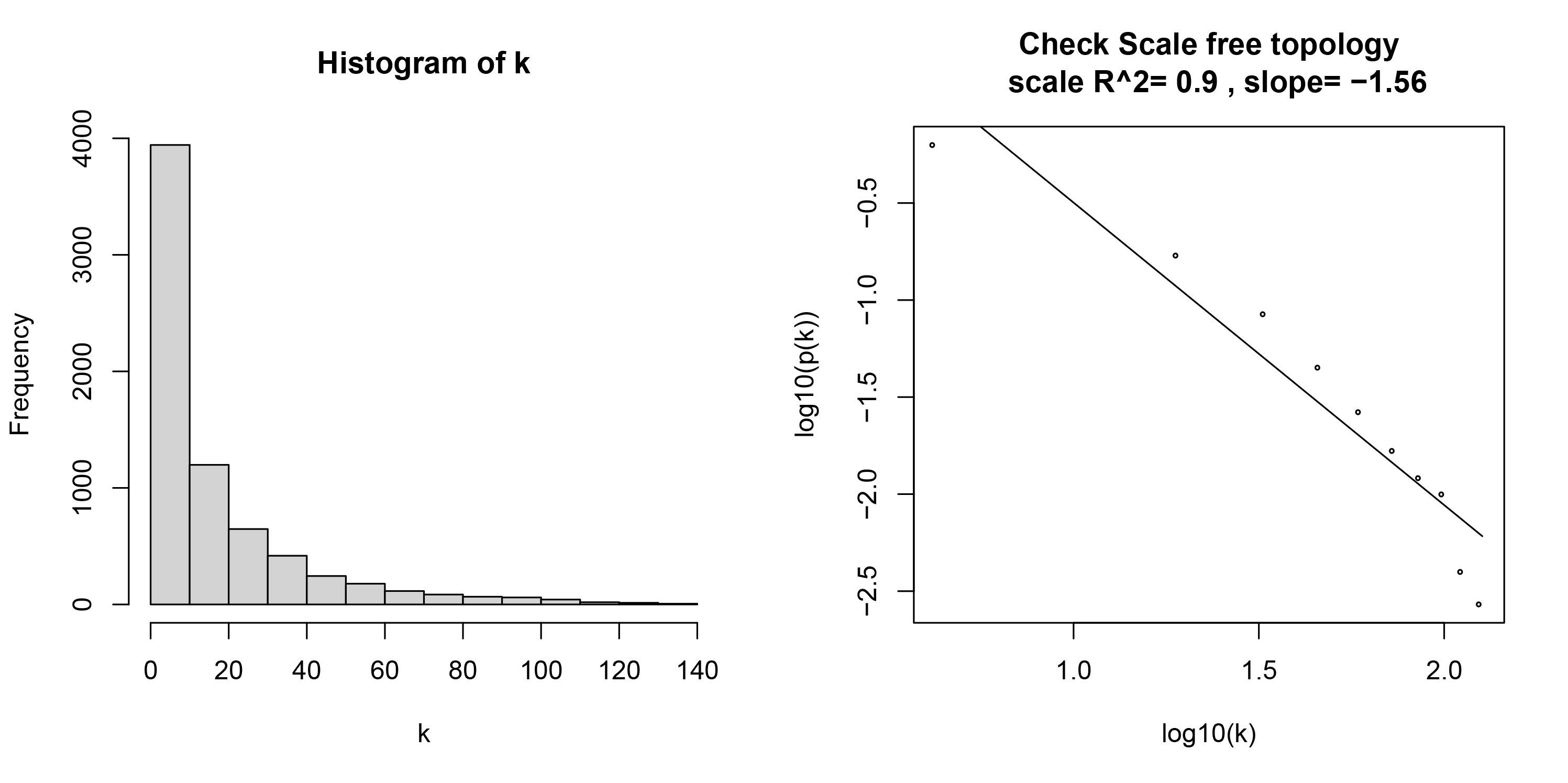

Supplement: Supplementary Figure 3 — The verification of the constructed scale-free network. R2 = 0.9; slope=-1.56. [file Image_3.tif]
