# Supplementary material for: Identification and Verification of Immune Subtype-Related lncRNAs in Clear Cell Renal Cell Carcinoma
Source: Front Oncol. 2022 Jun 2;12:888502. doi: 10.3389/fonc.2022.888502 (PMC9200973; doi:10.3389/fonc.2022.888502)
Supplement: Supplementary file 4 [file Table_1.docx]

| ID | Description | p-value | Count |
| --- | --- | --- | --- |
| hsa04660 | T cell receptor signaling pathway | <0.001 | 13 |
| hsa05340 | Primary immunodeficiency | <0.001 | 7 |
| hsa04658 | Th1 and Th2 cell differentiation | <0.001 | 9 |
| hsa04640 | Hematopoietic cell lineage | <0.001 | 9 |
| hsa04659 | Th17 cell differentiation | <0.001 | 9 |
| hsa04650 | Natural killer cell mediated cytotoxicity | <0.001 | 9 |
| hsa04514 | Cell adhesion molecules | <0.001 | 9 |
| hsa04060 | Cytokine-cytokine receptor interaction | <0.001 | 11 |
| hsa05235 | PD-L1 expression and PD-1 checkpoint pathway in cancer | <0.001 | 7 |
| hsa05142 | Chagas disease | <0.001 | 7 |

**Supplementary Table 1.** The enriched KEGG pathways by gene set enrichment analysis.

| ID | Description | pvalue | Count |
| --- | --- | --- | --- |
| GO:0042110 | T cell activation | <0.001 | 35 |
| GO:0050863 | regulation of T cell activation | <0.001 | 23 |
| GO:0030217 | T cell differentiation | <0.001 | 21 |
| GO:0030098 | lymphocyte differentiation | <0.001 | 22 |
| GO:1903131 | mononuclear cell differentiation | <0.001 | 22 |
| GO:0007159 | leukocyte cell-cell adhesion | <0.001 | 21 |
| GO:1903037 | regulation of leukocyte cell-cell adhesion | <0.001 | 19 |
| GO:0050870 | positive regulation of T cell activation | <0.001 | 15 |
| GO:0022407 | regulation of cell-cell adhesion | <0.001 | 19 |
| GO:0043368 | positive T cell selection | <0.001 | 9 |

**Supplementary Table 2.** The enriched biological process (BP) pathways by gene set enrichment analysis.
